# Sex‐specific vulnerability to depressive symptoms across adolescence and during the COVID‐19 pandemic: The role of the cingulum bundle

**DOI:** 10.1002/jcv2.12061

**Published:** 2022-02-24

**Authors:** Rajpreet Chahal, Tiffany C. Ho, Jonas G. Miller, Lauren R. Borchers, Ian H. Gotlib

**Affiliations:** ^1^ Department of Psychology Stanford University Stanford California USA; ^2^ Department of Psychiatry and Behavioral Science Weil Institute for Neuroscience University of California San Francisco California USA

**Keywords:** adolescence, cingulum, COVID‐19, depression, resilience, sex differences

## Abstract

**Background:**

Females are at higher risk for developing depression during adolescence than are males, particularly during exposure to stressors like the COVID‐19 pandemic. Examining structural connections between brain regions involved in executive functioning may advance our understanding of sex biases in stress and depression. Here, we examined the role of the cingulum bundle in differentiating trajectories of depressive symptoms in males and females across adolescence and during the pandemic.

**Methods:**

In a longitudinal study of 214 youth (121 females; ages 9–13 years at baseline), we examined whether fixel‐based properties of the cingulum bundle at baseline predict changes in females' and males' severity of depressive symptoms across four timepoints (4–7 years) in adolescence, including during the COVID‐19 pandemic. We also tested whether cingulum properties predict self‐reported resilience and stress during the pandemic.

**Results:**

Females had lower fiber density and cross‐section (FDC) of the cingulum than did males, a neural pattern that predicted greater increases in depressive symptoms, lower resilience, and higher stress during the COVID‐19 pandemic. Cingulum morphometry predicted changes in depressive trajectories in females, but not in males; specifically, females with lower FDC had significant increases in symptoms throughout adolescence, whereas females with higher cingulum FDC did not. Conversely, males had low, stable depressive symptoms throughout adolescence and higher resilience and lower stress during the pandemic compared to females. Higher cingulum FDC predicted higher resilience and lower stress in both sexes.

**Conclusions:**

In adults, the cingulum has been implicated in sex differences in stress reactivity. We show that in adolescents, the cingulum reflects sex differences in reports of stress and resilience that might contribute to the increased risk of stress‐related mood disorders in females. Adolescent females might benefit from cognitive interventions that strengthen the structural properties of the cingulum and increase their perceived resilience during periods of adversity and disruption.

## Graphical abstract

We examined the role of the cingulum bundle in predicting males’ and females’ changes in depressive symptom severity across four timepoints of adolescence, including during the COVID‐19 pandemic. Compared to males, cingulum fiber density and cross‐section (FDC) was lower in females; this lower pattern of FDC in females predicted increases in depressive symptoms across adolescence, as well as lower resilience and higher stress during the pandemic. The cingulum may be an important marker of sex differences in stress sensitivity and vulnerability for depression during adolescence.

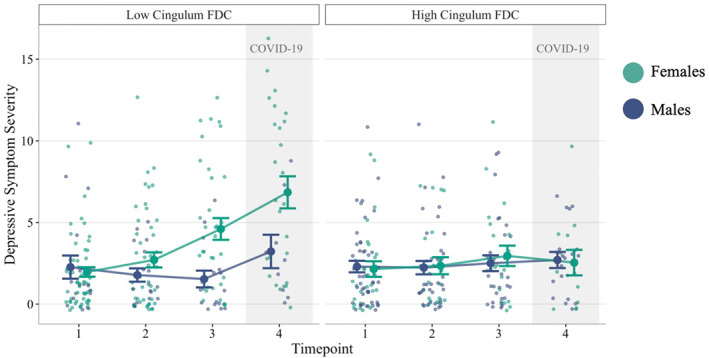

**Key points**

- The cingulum bundle connects fronto‐cingulate‐parietal brain regions involved in executive functioning; in adults, it has been implicated in resilience, depression, and sex differences in stress responsivity.
- We examined whether adolescent males' and females' cingulum morphometry predicts changes in depressive symptoms across four timepoints, including during the COVID‐19 pandemic.
- In early adolescence, males had higher cingulum fiber density and cross‐section (FDC) than did females, a pattern that predicted higher depressive symptoms, lower resilience, and higher stress during the pandemic.
- Only females with lower cingulum FDC increased in depressive symptoms throughout adolescence.
- The cingulum reflects sex differences in stress sensitivity and vulnerability for depression.

## INTRODUCTION

Depression is among the most prevalent of all mental health disorders, with the first emergence commonly occurring in adolescence (Bagalman & Cornell, 2018). Females are disproportionately affected by depression, particularly during the teenage years (Salk et al., 2017). The percentage of adolescent females with high levels of depressive symptoms appears to be growing in the past decade (Twenge, 2020); thus, it is critical that we take an interdisciplinary developmental perspective to gain a more comprehensive understanding of the etiology of sex differences in vulnerability to depression.

The most common environmental antecedent of depression is exposure to stressors (Kessler, 1997); neurobiological assessments may elucidate which adolescents are more or less sensitive to stress. While the impact of a stressor is often a function of its specific characteristics (e.g., duration, timing, severity), females have heightened stress sensitivity in the form of hypothalamic‐pituitary‐adrenal axis dysregulation and a higher incidence of stress‐induced mood disorders (Fernández‐Guasti et al., 2012). Relatedly, heightened stress responsivity in females has been found to increase during the pubertal phase, due in part to fluctuations in circulating gonadal hormones (Bale & Epperson, 2015). Neuroimaging studies also document sex differences in neural circuitry that may influence how stress dysregulation is programmed during adolescence (Colich et al., 2017), a sensitive period for both brain development and the onset of neuropsychiatric disorders (Juraska & Willing, 2017).

An important brain‐based correlate of adolescent depression is weakened fronto‐cingulate‐parietal neurocircuitry (Pan et al., 2020). Functional and structural connections among frontal, parietal, and temporal brain regions support executive control (Niendam et al., 2012), which is necessary for emotion regulation, particularly during periods of stress and uncertainty (Diamond, 2013). Functional and structural connections in these regions strengthen during adolescence (Sherman et al., 2014); further, connectivity of the fronto‐cingulate‐parietal executive control network in early adolescence has been found to predict psychological functioning in later adolescence (Jalbrzikowski et al., 2019). Similarly, we recently found that stronger functional connectivity within the executive control network buffered the risk of pubertally advanced youth for experiencing increases in internalizing symptoms during a period of significant stress – the COVID‐19 pandemic (Chahal, Kirshenbaum, et al., 2021). These neuroimaging‐based findings are consistent with behavioral evidence that executive functioning is impaired in adolescents with depression (Baune et al., 2014), and that stronger executive functioning buffers the adverse effects of risk factors for depression (Davidovich et al., 2016).

Importantly, females report more interpersonal stress during adolescence than do boys, which in turn predicts higher levels of negative coping styles (e.g., rumination) that are linked with the onset and maintenance of depression (Hamilton et al., 2015). Sex differences in neurobiology might also explain why females report having lower resilience and experience more adverse psychological consequences of stressful experiences than do males (Hodes & Epperson, 2019). Indeed, researchers have found sex differences in the neural signatures of executive functioning (e.g., higher cingulate activation in males during response inhibition; Gaillard et al., 2021) and resilience (e.g., higer correlation between resilience and orbitofrontal connectivity in males; Wang et al., 2020) in adolescence.

Although researchers have examined sex differences in neural *functional* signatures of resilience, it is unclear whether *structural* variations in white matter tracts related to executive functioning underlie sex differences in stress sensitivity and vulnerability to depression in adolescence. In particular, the cingulum bundle is a white matter association tract that connects prefrontal with temporal and parietal cortices (Agrawal et al., 2011). Microstructural strengthening of the cingulum has been shown to support improvements in executive functioning during adolescence (Bathelt et al., 2019). Further, lower fractional anisotropy (FA) of this tract has been found in young adults with higher depressive symptoms (Marečková et al., 2019), a neural signature that also underlies cognitive impairments in depressed patients (Schermuly et al., 2010). The cingulum bundle may also act as a marker of resilience, given that higher FA in this tract has been associated with higher levels of “grit” in a geriatric sample (Vlasova et al., 2018). Finally, adult males have higher FA of the cingulum bundle than do females, which is related to stress reactivity differently in men and women (Wheelock et al., 2021). Collectively, these findings suggest that microstructural properties of the cingulum bundle contribute to sex differences in stress reactivity, resilience, and vulnerability to depression.

In this study, we examined the cingulum bundle as a potential neural marker of risk for depressive symptoms over adolescence vulnerability during a period of stress. The SARS‐CoV‐2 (COVID‐19) pandemic has exacted a significant toll on adolescents' mental health, with many teens, particularly females, reporting increases in depressive symptoms (Barendse et al., 2021). We examined sex differences in morphometric properties of the cingulum bundle during early adolescence, and whether this tract predicts changes in depressive symptoms through adolescence. We also examined sex differences in the relation between the cingulum bundle and subjective resilience and perceived stress during the pandemic. Consistent with reports in adults (Wheelock et al., 2021), we hypothesized that, compared to males, females would exhibit lower micro‐ and macro‐structural metrics (i.e., a combined measure of fiber density and cross‐section (FDC) of the cingulum in early adolescence. We also expected that while males and females would not differ in depressive symptoms in early adolescence, females would exhibit steeper increases in symptoms over adolescence, reflecting a sex‐related divergence in risk for depression (Salk et al., 2017). Further, we expected that lower cingulum FDC in early adolescence would be associated with steeper increases in depressive symptoms through adolescence; since we expected steeper increases in depressive symptoms in females, we hypothesized that cingulum FDC would be more strongly associated with symptom changes in females as compared to males. That is, we expected depressive symptoms to remain relatively low in males throughout adolescence, thus we did not expect that cingulum FDC would track with variability in males' depressive symptoms. We also expected that lower cingulum FDC would be associated with higher perceived stress during the COVID‐19 pandemic, and with lower resilience during the pandemic, though we had no specific hypothesis about whether the relation between cingulum FDC and these constructs would differ for males and females.

## MATERIALS AND METHODS

### Participants

The sample included 214 children and young adolescents (121 females) who were recruited in September 2013 from the San Francisco Bay Area to participate in a study assessing the effects of early life stress on psychobiology across puberty. Participants were 9.11–13.98 years (*M* = 11.38 ± 1.05) at the first timepoint (T1). Because males and females were matched on pubertal stage, males (*M* = 11.81 ± 0.94) were slightly older than females (*M* = 11.06 ± 1.01; *t*(205) = 5.62, *p* < 0.01). Exclusion criteria included an inability to participate in the neuroimaging scan (e.g., non‐removable metal), intellectual delay, current or parent neurological disorders, non‐fluent English speakers, and self‐reported onset of menses for females (to ensure that participants were in early stages of puberty). 9% of the sample was Black, 8% Hispanic, 11% Asian, 21% Two or More Races, 45% White, and 6% other than what was listed. Males and females did not differ in racial and ethnic composition (*χ* ^2^ (5, *N* = 214) = 4.04, *p* = 0.540). The sample was of relatively high socioeconomic status based on parent‐reported highest education (0.47% no high school diploma/General Educational Development [GED]; 1.42% high school diploma/GED; 18.96% some college; 8.53% 2‐year college; 37.44% 4‐year college degree; 26.54% master's degree; 4.74% professional degree (e.g., MD, JD, DDS, doctorate); and 1.9% declined to answer) and income‐to‐needs ratios (*M* = 1.28 ± 0.56), calculated by dividing the caregiver‐reported total family income over the previous 12 months by the low‐income limit for Santa Clara County (King et al., 2019).

**FIGURE 1:**
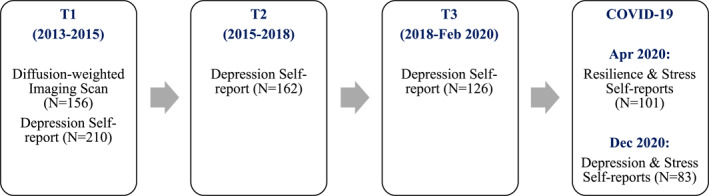
Flowchart of study data collection period. Whereas Table 2 shows the number of complete cases *across timepoints within each sex*, Figure 1 shows numbers *within timepoint across males and females*. Of the 121 females recruited in the study, 33 had four (28 with diffusion‐weighted imaging), 42 had three (31 with diffusion‐weighted imaging), 25 had two (19 with diffusion‐weighted imaging), and 21 had one (11 with diffusion‐weighted imaging) timepoint(s) of depressive symptom data = 254 complete cases (248 with reported pubertal stage and race group). Of the 93 males, 33 had four (27 with diffusion‐weighted imaging), 26 had three (20 with diffusion‐weighted imaging), 17 had two (12 with diffusion‐weighted imaging), and 17 had one (8 with diffusion‐weighted imaging) timepoint(s) of depressive symptom data = 200 complete cases (197 with reported pubertal stage and race group). The number of timepoints completed were not associated with sex, age, or cingulum FDC (all ps > 0.05)

Scan data were obtained at the first timepoint (T1 and participants completed questionnaires assessing their depressive symptoms across four timepoints. The average interval between T1 and T2 was 2.04 ± 0.39 years, and between T2 and T3 was 2.21 ± 0.53 years. In addition, in April of 2020, 2.5–4.5 weeks after the start of the March 2020 Bay Area shelter‐in‐place directive, participants completed a COVID‐19‐related survey in which we assessed self‐reported resilience (*N* = 101; approximately 1.08 ± 0.79 years after T3). Then, in December 2020, still during the shelter‐in‐place directive, participants completed another set of COVID‐19‐related surveys in which we assessed self‐reported depressive symptoms and perceived stress (*N* = 83). A flowchart of the data collection period is presented in Figure 1. The participants were relatively psychiatrically healthy with respect to diagnoses of Major Depressive Disorder (MDD) according to DSM‐IV criteria assessed with the Kiddie Schedule for Affective Disorders and Schizophrenia‐ Present and Lifetime version (K‐SADS‐PL; Kaufman et al., 1997). At T1, 1.40% of the participants had a past diagnosis of subthreshold MDD and no participants met criteria for a current diagnosis of MDD. At T2, 1.27% of participants met criteria for past MDD since their T1 visit, 0.63% met criteria for subthreshold MDD since their T1 visit, and 1.27% met criteria for current MDD. At T3, 5.00% of participants met criteria for past MDD since their last visit, 3.57% met criteria for subthreshold MDD since their last visit, and 7.14% met criteria for current MDD.

### Measures (T1‐T3 and COVID‐19 assessments)

*Depressive Symptoms (All Timepoints).* At T1‐T3 and during the December 2020 COVID‐19 assessment, participants completed the 10‐item version of the self‐reported Child Depression Inventory (Helsel & Matson, 1984). This widely used reliable measure (Saylor et al., 1984) has been shown to have convergent validity with clinician ratings of depression symptoms and diagnosis (Timbremont et al., 2004). Participants indicated the severity of symptoms of depression they were experiencing over the last 2 weeks on a three‐point scale, and a sum score was calculated (after appropriate reverse scoring). Cronbach's α was 0.75–0.88 at the four assessments.

*Resilience (April 2020 during COVID‐19).* Participants rated their ability to cope with adversity using the 10‐item Connor‐Davidson Resilience Scale (Connor & Davidson, 2003). This measure has excellent psychometric properties (Campbell‐Sills & Stein, 2007); in our sample, Cronbach's α was 0.90.

*Perceived Stress (April and December, 2020 of COVID‐19).* At both COVID‐19 assessments participants completed the 10‐item Perceived Stress Scale (Cohen et al., 1983), rating the degree to which they found their lives to be unpredictable, uncontrollable, and overloaded over the last month. The scale has acceptable psychometric properties (Lee, 2012) and in our sample Cronbach's α was 0.84–0.85.

*Pubertal Stage (T1).* Pubertal stage was assessed at T1 using the Tanner Staging Questionnaire (Morris & Udry, 1980), a measure shown to be significantly correlated with physician ratings of puberty‐related physical development (Shirtcliff et al., 2009). We averaged the Tanner pubic hair and breast/testes ratings to compute an index of overall pubertal development, as in previous work (Chahal, Delevich, et al., 2021).

### Diffusion imaging (T1)

*Acquisition.* Diffusion‐weighted neuroimaging data were obtained at the Stanford Center for Cognitive and Neurobiological Imaging (cni.stanford.edu). An echo planar imaging sequence was collected with 60 diffusion‐weighted directions and anterior‐to‐posterior phase encoding (echo time = 93.5 ms; repetition time = 8500 ms; voxel size = 0.938 × 0.938 × 2.00 mm; slices = 64; flip angle = 12°; *b* = 2000 mm^2^) (6 volumes *b* = 0). Nineteen participants were scanned using 2.00 mm^3^ voxel sizes after a scanner upgrade. To control for potential confounding effects of scan acquisition differences, we included a dichotomous covariate for scan acquisition group in our statistical models.

*Fixel‐based Analysis (FBA).* As described in previous work (Chahal, Delevich, et al., 2021), we used FBA to measure morphometric properties of white matter tracts. Fixel‐based Analysis applied higher‐order diffusion models to fiber populations within each voxel/fixel to estimate fiber density and fiber cross‐section per voxel. We combined these two measures to calculate combined FDC, allowing us to capture both micro‐ and macrostructural properties of white matter fibers. For sensitivity analyses, we also examined fiber density and fiber cross‐section separately. The FBA approach has been shown to be more sensitive to developmental changes, less sensitive to crossing fibers issues, and more interpretable than are standard voxel‐averaged quantitative measures of microstructure, including FA (Raffelt et al., 2017). We used MRtrix3 (Tournier et al., 2019) for diffusion‐weighted data processing, including: data denoising, eddy‐current induced distortion and motion correct, estimation of brain masks per individual, bias field correction to eliminate low‐frequency intensity inhomogeneities, and intensity normalization across the sample. Then, we performed the following steps: (1) estimation of a study‐specific white matter mask; (2) estimation of the group‐average response function; (3) up‐sampling of diffusion data and brain mask images; (4) estimation of fiber orientation distribution (FOD) using Constrained Spherical Deconvolution via the group average response function; (5) study‐specific FOD template generation; (6) registration of subject FOD images to the FOD template; (7) generation of white matter template fixel analysis mask; (8) thresholding of peak fixel image; (9) warping of FOD images to template space; (10) segmentation of FOD images to estimate fixels and their fiber density; (11) reorienting of fixel orientations in order to ensure that the subject and template fixels had angular correspondence; (12) assignment of subject fixel to template fixels; (13) computation of fiber cross‐section; and (14) computation of a combined measure of FDC (i.e., FDC). A full description of steps taken to compute FDC is available on the MRtrix website, along with documentation of commands (https://mrtrix.readthedocs.io/en/3.0_rc1/fixel_based_analysis/ss_fibre_density_cross‐section.html).

**FIGURE 2:**
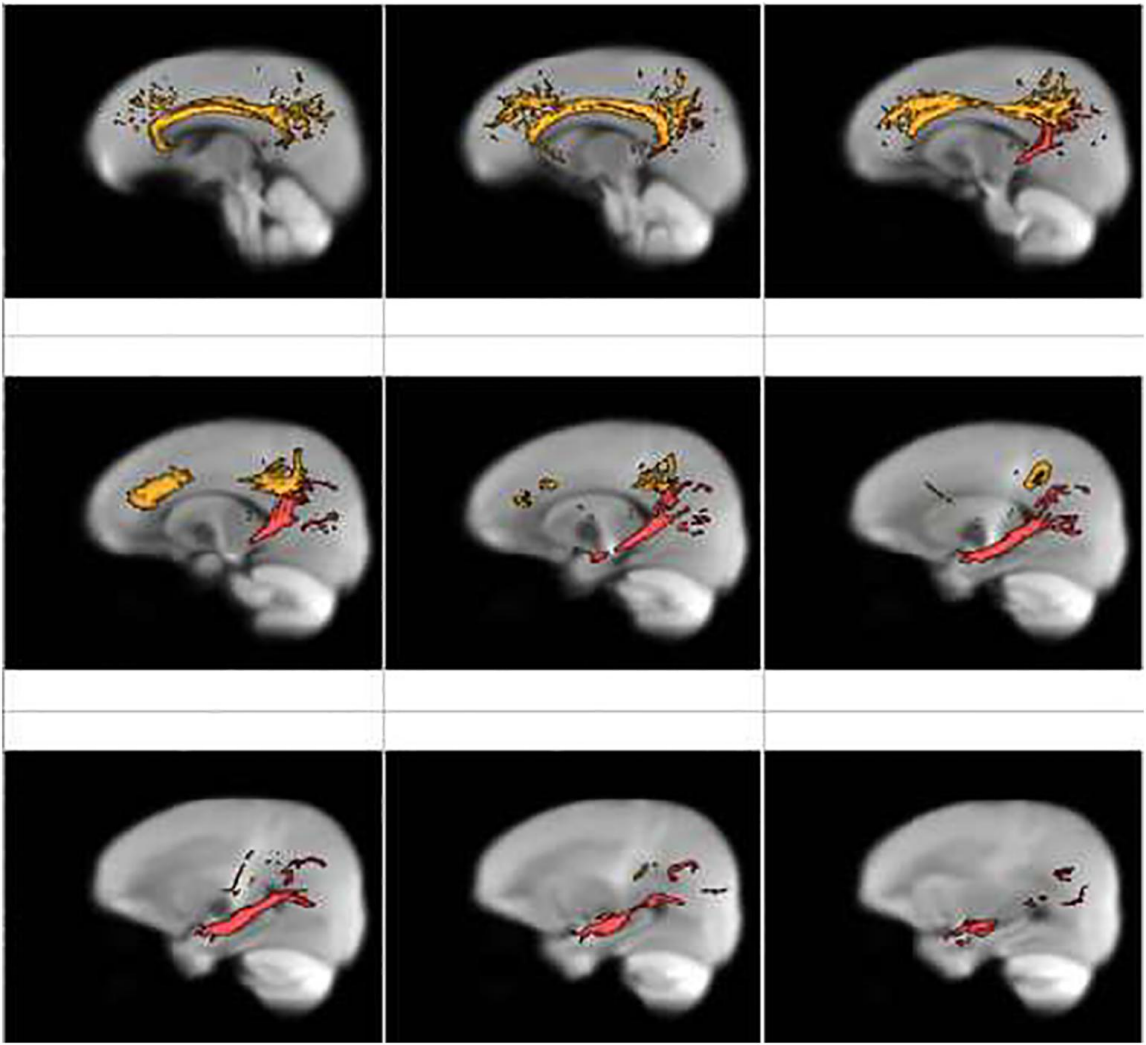
Cingulum segmentation masks overlaid on fiber orientation distribution (FOD) image. Cingulum masks from the Johns Hopkins University White‐Matter Labels Atlas are displayed. The cingulate portion is shown in orange and the hippocampal portion is shown in red

We used the Johns Hopkins University White‐Matter Labels Atlas, available through the FMRIB Software Library (Jenkinson et al., 2012), to extract per‐person estimates of the average FDC in the bilateral (average of left and right) cingulum bundle tracts (Figure 2). We combined the bilateral cingulate and hippocampal subdivisions of the cingulum in our main analyses; in follow‐up sensitivity analyses we also probed whether left or right and hippocampal or cingulate sub‐tracts contributed differentially to our findings. Finally, we extracted the bilateral average of the left and right frontoaccumbal tract (connecting orbitofrontal cortex and nucleus accumbens, as we described previously (Chahal, Delevich, et al., 2021), the uncinate fasciculus, and the superior longitudinal fasciculus (SLF) as control tracts in our follow‐up sensitivity analyses.

### The cingulum bundle and sex differences in depressive symptom trajectories, resilience, and perceived stress

Using *R* v. 3.6.1 (R Core Team, 2017), we first examined sex differences in severity of depressive symptoms and cingulum FDC at T1. Then, using the lme4 (Bates et al., 2021) and interactions (Long, 2021) packages in *R*, we conducted longitudinal multilevel modeling to test the main effect of cingulum FDC, the interaction of cingulum FDC and timepoint (0 = timepoint 1), and the 3‐way interaction of cingulum FDC, timepoint, and sex on trajectories of depressive symptom severity over the four timepoints (i.e., T1‐T3 and the December, 2020 COVID‐19 assessment). This analysis allowed us to test whether the cingulum bundle is related to depressive symptom trajectories differently in males and females. We conducted separate follow‐up models in males and females for simple slopes analyses in two‐way interactions (timepoint x cingulum FDC). We used timepoint rather than age as the main effect of interest given that we wanted to examine changes in depressive symptoms from pre‐to during the pandemic, and age at each timepoint varied across the sample. We included age at all timepoints and T1 pubertal stage, race, and scan acquisition group as covariates in longitudinal models. We conducted analyses on complete cases (i.e., participants for whom all variables of interest were available within a given timepoint, including severity of depressive symptoms, cingulum FDC, pubertal stage, and race) using restricted maximum likelihood estimation, which utilizes available complete data (i.e., within a timepoint) even if fewer than four timepoints of data were available for a given individual. Further information about the number of cases included in the model are included in Table 2 and Figure 1.

Unlike the above longitudinal analysis of symptoms across adolescence, we also used repeated measures analysis of variance to examine whether changes in depressive symptoms from pre‐to during the pandemic were associated with cingulum FDC in males and females in a combined model that we then probed using follow‐up simple slopes analyses within each sex if the interaction of sex and cingulum FDC was significant. We also conducted multiple regressions to test whether there were significant associations between cingulum FDC and resilience and perceived stress during the pandemic in both males and females in combined models. If interactions between sex and cingulum FDC were present, we probed sex differences further using simple slopes analyses. All variables (i.e., depressive symptom severity, resilience, perceived stress, cingulum FDC) were standardized within sex.

As we noted above, we conducted follow‐up sensitivity analyses in which we tested whether specific aspects of cingulum FDC (left or right laterality; cingulate or hippocampal subdivisions) contributed to our findings. Finally, we also tested whether the findings were specific to the cingulum or were also evident using FDC of three other tracts involved in aspects of cognitive and emotional functioning: the frontoaccumbal tract (a reward‐related white matter pathway); the uncinate fasciculus (a socio‐emotional processing tract connecting the inferior frontal lobe with limbic regions); and the SLF (a language and cognitive processing long‐range lateral tract that, like the cingulum, connects frontal and parietal lobes).

## RESULTS

### Participants and sample characteristics

Sample characteristics and correlations among variables are presented in Table 1. Cingulum FDC was positively associated with age at T1‐T3 (*p*s < 0.01), but not with depressive symptom severity at T1‐T3 in either males or females (*p*s > 0.05). However, during the pandemic, higher cingulum FDC was associated with lower depressive symptom severity and perceived stress, and with higher resilience (all *p*s < .05). Resilience was negatively associated with depressive symptom severity at all timepoints (*p*s < 0.01).

**TABLE 1:** Means, standard deviations, and correlations with confidence intervals

Variable	*M*	*SD*	1	2	3	4	5	6	7	8	9	10	11	12
1. T1 Age (*N* = 214)	11.38	1.05												
2. T2 Age (*N* = 164)	13.39	1.07	0.93**											
			[0.91, 0.95]											
3. T3 Age (*N* = 129)	15.57	1.16	0.84**	0.88**										
			[0.79, 0.88]	[0.83, 0.91]										
4. COVID‐19 (Apr) Age (*N* = 101)	16.51	1.36	0.84**	0.79**	0.81**									
			[0.77, 0.89]	[0.70, 0.85]	[0.72, 0.87]									
5. COVID‐19 (Dec) Age (*N* = 83)	17.07	1.36	0.85**	0.78**	0.79**	0.99**								
			[0.77, 0.90]	[0.68, 0.86]	[0.69, 0.86]	[0.98, 0.99]								
6. T1 Cingulum FDC (*N* = 156)	0.30	0.03	0.28**	0.24**	0.26**	0.13	0.10							
			[0.13, 0.42]	[0.07, 0.40]	[0.09, 0.42]	[−0.08, 0.34]	[−0.14, 0.33]							
7. T1 Depressive severity (*N* = 210)	2.20	2.40	0.02	0.04	−0.12	−0.09	−0.11	0.00						
			[−0.12, 0.15]	[−0.12, 0.19]	[−0.28, 0.04]	[−0.28, 0.11]	[−0.32, 0.11]	[−0.16, 0.16]						
8. T2 Depressive severity (*N* = 162)	2.38	2.64	0.09	0.10	0.04	0.14	0.11	−0.07	0.29**					
			[−0.06, 0.24]	[−0.06, 0.25]	[−0.13, 0.21]	[−0.06, 0.34]	[−0.12, 0.33]	[−0.24, 0.11]	[0.15, 0.43]					
9. T3 Depressive severity (*N* = 126)	3.53	3.47	−0.14	−0.12	−0.02	0.01	−0.03	−0.08	0.08	0.60**				
			[−0.30, 0.02]	[−0.28, 0.06]	[−0.18, 0.14]	[−0.19, 0.21]	[−0.24, 0.20]	[−0.26, 0.10]	[−0.08, 0.24]	[0.48, 0.70]				
10. COVID‐19 (Dec) depressive severity (*N* = 83)	4.39	4.17	−0.19	−0.10	−0.16	−0.10	−0.09	−0.35**	0.22*	0.49**	0.44**			
			[−0.39, 0.03]	[−0.32, 0.12]	[−0.37, 0.06]	[−0.30, 0.12]	[−0.30, 0.12]	[−0.54, −0.12]	[0.01, 0.42]	[0.30, 0.65]	[0.25, 0.60]			
11. COVID‐19 (Apr) resilience score (*N* = 101)	27.12	7.13	0.22*	0.20	0.16	0.12	0.19	0.28**	−0.29**	−0.30**	−0.30**	−0.55**		
			[0.03, 0.40]	[−0.01, 0.39]	[−0.04, 0.35]	[−0.08, 0.31]	[−0.03, 0.39]	[0.07, 0.47]	[−0.46, −0.10]	[−0.47, −0.10]	[−0.47, −0.10]	[−0.68, −0.38]		
12. COVID‐19 (Apr) perceived stress (*N* = 101)	18.60	6.66	−0.07	−0.08	0.00	0.09	−0.02	−0.27*	0.22*	0.24*	0.33**	0.44**	−0.36**	
			[−0.26, 0.13]	[−0.28, 0.13]	[−0.21, 0.20]	[−0.11, 0.28]	[−0.23, 0.20]	[−0.46, −0.06]	[0.03, 0.40]	[0.03, 0.42]	[0.13, 0.50]	[0.24, 0.60]	[−0.52, −0.17]	
13. COVID‐19 (Dec) perceived stress (*N* = 83)	16.96	6.65	−0.13	−0.07	−0.14	−0.03	−0.04	−0.30*	0.17	0.41**	0.34**	0.71**	−0.41**	0.54**
			[−0.34, 0.09]	[−0.29, 0.16]	[−0.35, 0.08]	[−0.25, 0.19]	[−0.26, 0.18]	[−0.50, −0.06]	[−0.05, 0.37]	[0.20, 0.58]	[0.12, 0.52]	[0.59, 0.81]	[−0.58, −0.21]	[0.37, 0.68]

*Note:* Values in square brackets indicate the 95% confidence interval for each correlation.

Abbreviation: FDC, fiber density and cross‐section.

*p < 0.05. **p < 0.01.

### Sex differences in cingulum morphometry

At T1, females (*M* = 0.29) had lower cingulum FDC than did males (*M* = 0.31; *t*(125.05) = 5.00, *p* < 0.001), even when controlling for age, scan acquisition group, pubertal stage, race, and depressive symptom severity at T1 (*β* = −0.32, 95% confidence interval (CI) [−0.33 to −0.31], *t*(134) = −3.86, *p* < 0.001).

### Cingulum morphometry and longitudinal changes in depressive symptoms

A full‐sample longitudinal multilevel model, with a random intercept and fixed slope (though varying by sex and cingulum FDC) yielded a significant three‐way interaction of timepoint, sex, and cingulum FDC (*β* = 0.10 [0.02–0.19], *t*(354.27) = 2.50, *p* = 0.012). Including a random slope term led to failed model convergence issues due to overfitting (i.e., too many random effects).

**FIGURE 3:**
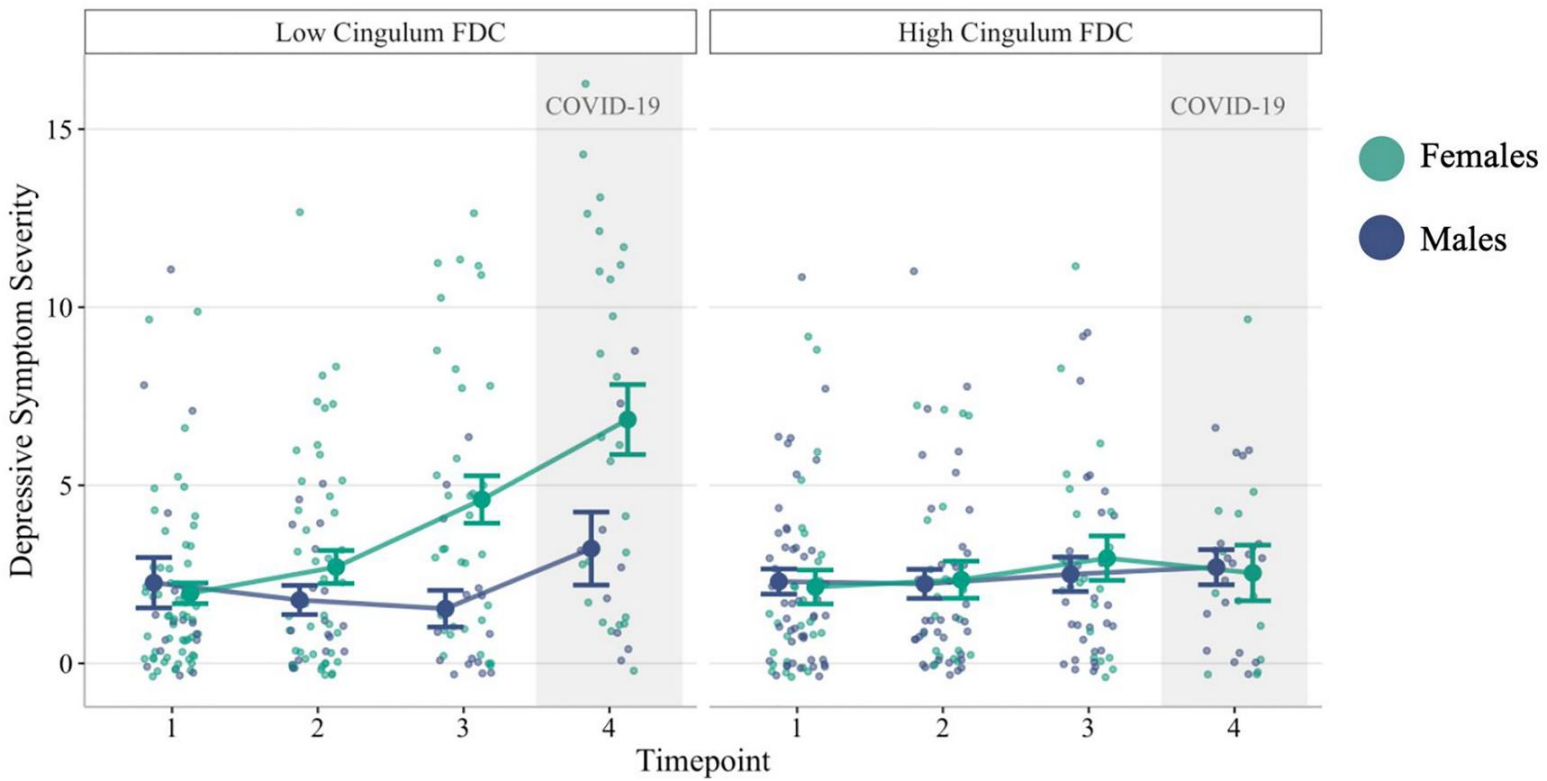
Longitudinal changes in depressive symptom severity: The roles of cingulum morphometry and sex differences. Timepoint 4 was the December, 2020 COVID‐19 assessment. Cingulum FDC is only grouped for visualization (Mean ‐ 1SD = low, Mean + 1SD = high). FDC, fiber density and cross‐section

We then conducted two models testing the interaction of timepoint and cingulum FDC in males and females separately for ease of interpreting simple slopes analyses and coefficient extractions. In males, no variable (including cingulum FDC) was significantly associated with changes in the severity of depressive symptoms (*p*s > 0.05). In contrast, in females, the main effect of timepoint (*β* = 0.41 [0.10–0.72], *t*(153.36) = 2.59, *p* = 0.010) and the interaction of cingulum FDC and timepoint (*β* = −0.17 [−0.30 to −0.04], *t*(198.57) = −2.62, *p* = 0.009) were significant. Follow‐up simple slopes analyses indicated that females with lower cingulum FDC (1SD below the mean) had increasing severity of depressive symptoms over time (*β* = 0.60 [0.28–0.93], *t(99.29)* = 3.62, *p* < 0.001); in contrast, females with higher cingulum FDC (mean + 1SD) did not change significantly in symptoms across time (*p* > 0.05). We also probed whether changes in depressive symptoms across specific timepoints were driving our findings. In females, increases in depressive symptoms were significant between T1 and T3, T1 and COVID‐19, T2 and T3, T2 and COVID‐19, and between T3 and COVID‐19, but only when cingulum FDC was lower (all *p*s < 0.01); no changes in females' severity of depressive symptoms across adolescence were significant when cingulum FDC was higher (all *p*s > 0.05; Table 2; Figure 3). Follow‐up tests and plots showed that all assumptions of multilevel models were met, including linearity, homogeneity of variance (Levene's test *p* > 0.05), and normal distribution of model residuals.

**TABLE 2:** Sex differences in the association between cingulum morphometry and longitudinal changes in depressive symptom severity across four timepoints

*Predictors*	Full sample model			Males only model			Females only model		
	*Estimates*	*CI*	*p*	*Estimates*	*CI*	*p*	*Estimates*	*CI*	*p*
Intercept	−0.39	−0.90–0.11	0.128	−0.30	−0.90–0.30	0.323	−0.47	−1.25–0.32	0.243
Timepoint	0.17	−0.05–0.39	0.121	−0.20	−0.50–0.10	0.199	0.41	0.10–0.72	**0.010**
Cingulum FDC	0.01	−0.15–0.16	0.922	−0.05	−0.22–0.13	0.622	0.03	−0.22–0.29	0.801
Age	0.02	−0.23–0.27	0.885	0.32	−0.02–0.66	0.069	−0.11	−0.46–0.23	0.520
Scan group	−0.10	−0.29–0.08	0.279	0.05	−0.17–0.28	0.641	−0.21	−0.49–0.07	0.146
Pubertal stage (T1)	0.11	−0.06–0.28	0.189	0.15	−0.08–0.37	0.192	0.10	−0.13–0.34	0.393
Race group	0.02	−0.09–0.14	0.695	0.06	−0.08–0.20	0.401	0.00	−0.17–0.16	0.986
Sex	0.04	−0.11–0.20	0.591						
Timepoint *x* cingulum FDC	−0.07	−0.15–0.01	0.099	0.04	−0.06–0.14	0.425	−0.17	−0.30 to −0.04	**0.009**
Timepoint *x* sex	−0.12	−0.20 to −0.04	**0.002**						
Sex *x* cingulum FDC	−0.02	−0.17–0.14	0.828						
Timpoint *x* sex *x* cingulum FDC	0.10	0.02–0.19	**0.012**						
**Random effects**									
σ2		0.66			0.49			0.8	
τ00		0.24			0.15			0.29	
ICC		0.27			0.23			0.27	
N		156			67			89	
Observations		445			197			248	
Marginal R2/Conditional R2		0.136/0.366			0.062/0.281			0.166/0.388	

*Note*: *p* < 0.05 is indicated in bold. All models included a random term for intercept. The number of observations reported are the number of observations with all variables of interest (i.e., complete cases including diffusion‐weighted imaging, reported pubertal stage and race group, and depressive symptom severity across timepoints); 445 observations across four timepoints were included in the full sample model, 197 in the male‐only model, and 248 in the female‐only model.

Abbreviations: CI, confidence interval; FDC, fiber density and cross‐section; ICC, intraclass correlation coefficient.

### Sex differences in depressive symptoms, resilience, and perceived stress during the COVID‐19 pandemic: The role of the cingulum bundle

Compared to males, females had higher depressive symptom severity (*M*_Females_ = 5.42, *M*_Males_ = 3.03 in December, 2020), lower resilience (*M*_Females_ = 24.97, *M*_Males_ = 30.02 in April, 2020), and higher perceived stress (*M*_Females_ = 20.74, *M*_Males_ = 15.64 in April, 2020; *M*_Females_ = 18.89, *M*_Males_ = 14.50 in December, 2020) during the COVID‐19 pandemic (*t*s = −2.90, −4.08, −3.14, and 3.85, respectively, *p*s < 0.003).

Paired sample *t*‐tests revealed that from T3 (the assessment closest in time to the COVID‐19 assessment) to the December, 2020 COVID‐19 timepoint, depressive symptom severity increased for females (*b* = 1.78 [0.08–3.48], *t*(36) = 2.13, *p* = 0.040), but not for males (*p* > 0.05). Importantly, a repeated measures analysis of variance including only the T3 and COVID‐19 depressive symptom data yielded a significant interaction of sex and cingulum FDC (*F* (1, 103) = 6.15, *p* = 0.015), as well as an interaction of timepoint and cingulum FDC (*F* (1, 103) = 5.19, *p* = 0.025). Follow‐up analyses indicated that whereas for females higher cingulum FDC (mean + 1 SD) was associated with lower depressive symptom severity at both timepoints (pre‐ and during the pandemic; *β* = −0.32 [−0.55 to −0.08], *t*(58) = −2.58, *p* = 0.011), for males cingulum FDC was not related to symptoms (*p* > 0.05). Further, participants with lower cingulum FDC (mean − 1 SD) increased significantly in severity of depressive symptoms from pre‐to during the pandemic (*β* = 0.60 [0.15–1.06], *t*(84.5) = 2.60, *p* = 0.010), while participants with higher cingulum FDC did not change in symptom levels (*p* > 0.05). We did not probe the 3‐way interaction of timepoint, cingulum FDC, and sex given that it was not significant.

Finally, while cingulum FDC was negatively associated with depressive symptom severity during the pandemic in females (*β* = −0.35 [−0.64 to −0.06], *t*(37) = −2.41, *p* = 0.021), this was not the case in males (*p* > 0.05). However, males and females did not differ in the associations between cingulum FDC and perceived stress or resilience during the pandemic: in both sexes there was a positive relation between cingulum FDC and resilience (*β* = 0.39 [0.06–0.71], *t*(80) = 2.34, *p* = 0.021) and a negative relation between cingulum FDC and perceived stress (*β* = −0.29 [−0.51 to −0.06], *t*(79) = −2.49, *p* = 0.015).

### Sensitivity analyses

Males and females differed in cingulum fiber cross‐section (*p* < 0.001), but not density (*p* > 0.05). There were sex differences for both the cingulate and hippocampal portions of the cingulum bundle (*p*s < 0.001), and both the left and right portions of these tracts (*p*s < 0.001). Similarly, associations between cingulum fiber *cross‐section* and longitudinal changes in depressive symptoms severity in females were significant (*p* = 0.029), but the relation with cingulum fiber *density* was not (*p* > 0.05). Finally, the associations between cingulum fiber cross‐section and depressive symptom severity, resilience, and perceived stress at COVID‐19 assessments were significant (*p*s < 0.02), but relations with fiber density were not (*p*s > 0.05).

Males had higher FDC in the frontoaccumbal tract, uncinate fasciculus, and SLF than did females (all *p*s < 0.01). The frontoaccumbal tract and uncinate fasciculus were not associated with changes in the severity of depressive symptoms or with depressive symptom severity, resilience, or perceived stress during the pandemic (all *p*s > 0.05). However, SLF FDC was associated with changes in depressive symptoms in females in the same manner as the cingulum; that is, the interaction of SLF FDC and timepoint was significant (*β* = −0.16 [−0.27 to −0.05], *t*(201.60) = −2.84, *p* = 0.005). Follow‐up simple slopes analyses indicated that females with lower SLF FDC (mean – 1 SD) had increasing severity of depressive symptoms over time (*β* = 0.56 [0.27–0.86], *t*(100.5) = 3.70, *p* < 0.001); in contrast, females with higher SLF FDC (mean + 1 SD) did not change significantly in symptoms across time (*p* > 0.05). In males, SLF FDC was not associated with changes in depressive symptoms. Like the cingulum, FDC of the SLF was negatively associated with the severity of depressive symptoms during the pandemic in females (*β* = −0.65 [−1.01 to −0.29], *t*(44) = −3.62, *p* < 0.001), but not in males (*p* > 0.05). Finally, males and females did not differ in the associations between SLF FDC and perceived stress or resilience during the pandemic: in both sexes there was a positive association between SLF FDC and resilience (*β* = 0.26 [0.03–0.49], *t*(77) = 2.28, *p* = 0.025) and a negative association between SLF FDC and perceived stress (*β* = −0.30 [−0.54 to −0.06], *t*(77) = −2.47, *p* = 0.016).

## DISCUSSION

In a community sample, we explored the role of the cingulum bundle in predicting sex differences in trajectories of depressive symptoms throughout adolescence, including during a period of significant stress – the COVID‐19 pandemic. In early adolescence, females had lower FDC of the cingulum than did males, a neural signature associated with greater increases in depressive symptoms, lower resilience, and higher perceived stress during the pandemic. Further, lower cingulum FDC in females was associated with significant increases in the severity of depressive symptoms throughout adolescence; in contrast, females with higher cingulum FDC had stable, low symptom severity over time. Overall, males remained relatively low in depressive severity throughout adolescence and showed no significant increase in symptom severity from pre‐to during the pandemic. Compared to females, males also had significantly lower severity of depressive symptoms, higher resilience, and lower perceived stress during the pandemic. Importantly, however, in both males and females there was a positive association between cingulum (and SLF) FDC and resilience, and a negative association between cingulum (and SLF) FDC and perceived stress during the pandemic. This suggests that while altered fronto‐parietal white matter morphometry predicts increasing trajectories of females' depressive symptoms in adolescence, these tracts also may reflect perceptions about both boys' and girls' stress sensitivity. Although these tracts did not predict trajectories of depressive symptoms in males, they were associated with stress and resilience in males. Low levels of endorsements of depressive symptoms in males may contribute to these findings; indeed, in a less psychiatrically healthy sample we might have also found that fronto‐parietal white matter morphometry identifies adolescents, regardless of sex, who are prone to experience mood dysregulation, particularly during stressful periods.

We recently showed in this sample that higher functional connectivity in the executive control network during early adolescence buffers puberty‐related increases in internalizing symptoms from pre‐to during the COVID‐19 pandemic (Chahal, Kirshenbaum, et al., 2021). The cingulum bundle connects fronto‐cingulate‐parietal brain regions that constitute many of the regions in the executive control network that are involved in executive functioning (Bathelt et al., 2019). Higher FDC, which captures both micro‐ and macrostructural properties such as axonal diameter and volume, in the cingulum may contribute to more efficient neuronal signaling among distant prefrontal, cingulate, temporal, and parietal regions required to support cognitive domains of emotion regulation, particularly during periods of stress (Schermuly et al., 2010). Studies suggest that cingulum morphometry and executive functioning abilities that contribute to emotion regulation continue to develop during adolescence and young adulthood (Bathelt et al., 2019); thus, since resilience is malleable, interventions and training programs aimed at increasing resilience by targeting these neurocognitive systems may have long‐term positive effects on individuals' coping with stress (Joyce et al., 2018), particularly for adolescent females who are at heightened risk for developing depression (Salk et al., 2017).

We found that females had lower FDC in the cingulum at T1, even after controlling for age differences between the sexes. This is consistent with prior research in adults showing that males have higher FA in the cingulum. Given evidence that microstructural properties of the tract are related to stress sensitivity differently in males and females (Wheelock et al., 2021), it is possible that lower morphometric cingulum properties in females contribute to or reflect sex differences in executive functioning in the context of stress. This is not to say that females have lower emotion regulatory abilities in general (e.g., due to sex‐linked genetic differences); rather, females experience greater interpersonal stressors (Hamilton et al., 2015) that impair their executive functioning (Shields et al., 2016) and increase their likelihood of developing internalizing forms of psychopathology, such as depression (Salk et al., 2017). This argument is supported by a meta‐analysis showing that sex differences are not detected in executive functioning tasks in the absence of stressors (Grissom & Reyes, 2019), and that stressors may negatively affect cognitive performance in females more so than in males (Yoon et al., 2009). The potential role of the cingulum in stress responsivity is also supported by our finding that higher cingulum FDC was related to lower severity of depressive symptoms, higher resilience, and lower stress during the COVID‐19 pandemic; however, morphometric properties of this tract were not related to depressive symptom severity during the timepoints prior to COVID‐19. Further, tract morphometry was associated with perceived stress and self‐reported resilience, both of which reflect individuals' coping with stressors.

Males may have faster expansion of certain white matter tracts during adolescence, and our finding of sex differences in cingulum properties should be replicated using a longitudinal neuroimaging sample. Indeed, we have previously found higher frontoaccumbal tract FDC in males than in females (Chahal, Delevich, et al., 2021), suggesting that this sex difference in FDC is not specific to the cingulum bundle. This interpretation is also consistent with prior work showing that while fiber cross‐section expansion occurs for all adolescents during pubertal development, males have larger white matter volume compared to females (Brouwer et al., 2012). It is possible that later in development, females may catch up to males in cingulum FDC, or that differences in this tract persist throughout adulthood and represent sex‐specific neural markers of stress sensitivity (Wheelock et al., 2021).

Our follow‐up analyses indicated that the cingulum may not be the only white matter tract that is related to the constructs of stress sensitivity and resilience. We found that the SLF, another tract connecting frontal and posterior parietal regions that has been implicated in cognitive control (Frye et al., 2010; Vestergaard et al., 2011), also predicted changes in the severity of depressive symptoms throughout adolescence and during the pandemic in females, as well as perceived stress and resilience during the pandemic in males and females. Consistent with our prior findings that functional connections among fronto‐parietal regions are associated with resilience to internalizing symptoms (Chahal, Kirshenbaum, et al., 2021), resilience to depression symptoms and stress may be more strongly related to structural connections among frontal and parietal regions that subserve cognitive functions than they are to pathways among frontal and limbic regions (i.e., the frontoaccumbal tract and uncinate fasciculus) that undergird reward or socio‐emotional processing. Thus, although in the present study we focused primarily on the cingulum bundle, other fronto‐parietal pathways may also be candidate markers of sex differences in vulnerability to depression.

We should note three limitations of this study. First, we did not administer explicit measures of executive functioning, limiting our ability to draw conclusions about the purported role of the cingulum bundle in emotion regulation and resilience. Second, we did not analyze neuroimaging data longitudinally in the present study to assess whether the cingulum develops differently in males and females, or the directional associations among cingulum morphometry, depressive symptoms, resilience, and stress perception. In addition, we were not able to assess whether adolescents met interviewer‐assessed diagnostic criteria for depression during the pandemic because the assessment at this timepoint was conducted with questionnaires administered online. Finally, we had attrition in our sample, particularly in the COVID‐19 assessments, when we had a 50% response rate compared to prior timepoints. The COVID‐19 pandemic was a difficult event for most individuals and particularly for adolescents, given the marked changes in academic, social, and familial environments (Barendse et al., 2021). Although we expected to have lower response rates during the pandemic, we recognize the potential impact on our findings.

## CONCLUSION

The cingulum bundle connects frontal, cingulate, parietal, and temporal regions of the brain involved in executive processing and emotion regulation. While limited previous research in adults suggests that higher microstructural properties of the cingulum are related to higher measures of resilience and lower stress sensitivity, our study is the first to show that FDC of the cingulum predicts changes in depressive symptoms throughout adolescence, particularly during extended periods of significant stress and uncertainty. Importantly, we observed that females had lower FDC of the cingulum than did males, a neural signature that predicted greater increases in depressive symptoms, lower resilience, and higher stress during the pandemic. Our finding that the cingulum was associated with changes in depressive symptoms throughout adolescence only in females might be due to the relatively low stable levels of symptoms in males in this sample. Although we did not measure executive functioning, we do not expect that sex differences in executive functioning would explain the female bias in susceptibility to depression during adolescence. Rather, we expect that females experience more stressors and internalize stress more strongly than do males, and that the cingulum bundle reflects sex differences in stress sensitivity and vulnerability for depression. Future studies examining changes in the cingulum and in symptoms of depression in adolescents who exhibit wider variability in stress responses and symptomatology (including those with clinical disorders) will be important for confirming that the relation between this tract and stress sensitivity is different in males and females.

## CONFLICT OF INTERESTS

The authors have declared that they have no competing or potential conflicts of interest.

## ETHICAL CONSIDERATION

All adolescents and their parent(s)/legal guardian(s) provided informed assent and consent, respectively. This study was approved by the Stanford University Institutional Review Board.

## AUTHOR CONTRIBUTION

**Rajpreet Chahal:** Conceptualization; Data curation, Formal analysis; Investigation; Methodology; Software; Validation; Visualization; Writing – original draft; Writing – review & editing. **Tiffany Ho:** Methodology; Validation; Writing – review & editing. **Jonas Miller:** Validation; Writing – review & editing. **Lauren R. Borchers:** Writing – review & editing. **Ian H. Gotlib:** Funding acquisition; Project administration; Resources; Supervision; Validation; Writing – review & editing.

### OPEN PRACTICES BADGES

This article has earned an Open Data badge for making publicly available the digitally‐shareable data necessary to reproduce the reported results. The data is available at https://nda.nih.gov/.

## Data Availability

Raw data were generated at the Stanford Center for Cognitive and Neurobiological Imaging and at the Department of Psychology at Stanford University. The data that support the findings of this study are available in the National Institute of Mental Health Data Archive (NDA) at https://nda.nih.gov/. The project is 4R01MH101495‐04 and the NDA Collection ID is C2135. Third party software information and versions are also detailed in the manuscript.

## References

[jcv212061-bib-0001] Agrawal, A. , Kapfhammer, J. P. , Kress, A. , Wichers, H. , Deep, A. , Feindel, W. , Sonntag, V. K. H. , Spetzler, R. F. , & Preul, M. C. (2011). Josef Klingler’s models of white matter tracts: Influences on neuroanatomy, neurosurgery, and neuroimaging. Neurosurgery, 69(2), 238–254. discussion 252‐254. 10.1227/NEU.0b013e318214ab79 21368687

[jcv212061-bib-0002] Bagalman, E. , & Cornell, A. (2018). Prevalence of mental illness in the United States: Data sources and estimates (Vol. 11). Congressional Resaerch Service.

[jcv212061-bib-0003] Bale, T. L. , & Epperson, C. N. (2015). Sex differences and stress across the lifespan. Nature Neuroscience, 18(10), 1413–1420. 10.1038/nn.4112 26404716 PMC4620712

[jcv212061-bib-0004] Barendse, M. , Flannery, J. , Cavanagh, C. , Aristizabal, M. , Becker, S. P. , Berger, E. , Breaux, R. , Campione‐Barr, N. , Church, J. A. , Crone, E. , Dahl, R. , Dennis‐Tiwary, T. A. , Dvorsky, M. , Dziura, S. L. , van de Groep, S. , Ho, T. , Killoren, S. E. , Langberg, J. M. , Larguinho, T. , … Pfeifer, J. H. (2021). Longitudinal change in adolescent depression and anxiety symptoms from before to during the COVID‐19 pandemic: An international collaborative of 12 samples. PsyArXiv. 10.31234/osf.io/hn7us PMC9349954 35799311

[jcv212061-bib-0005] Bates, D. , Maechler, M. , Bolker, B. , Walker, S. , Christensen, R. H. B. , Singmann, H. , Dai, B. , Scheipl, F. , Grothendieck, G. , Green, P. , Fox, J. , Bauer, A. , & Krivitsky, P. N. (2021). lme4: Linear Mixed‐Effects Models using “Eigen” and S4 (Version 1.1‐27.1). Retrieved from https://CRAN.R‐project.org/package=lme4

[jcv212061-bib-0006] Bathelt, J. , Johnson, A. , Zhang, M. , & Astle, D. E. (2019). The cingulum as a marker of individual differences in neurocognitive development. Scientific Reports, 9(1), 2281. 10.1038/s41598-019-38894-z 30783161 PMC6381161

[jcv212061-bib-0007] Baune, B. T. , Fuhr, M. , Air, T. , & Hering, C. (2014). Neuropsychological functioning in adolescents and young adults with major depressive disorder – a review. Psychiatry Research, 218(3), 261–271. 10.1016/j.psychres.2014.04.052 24851725

[jcv212061-bib-0008] Brouwer, R. M. , Mandl, R. C. W. , Schnack, H. G. , van Soelen, I. L. C. , van Baal, G. C. , Peper, J. S. , Kahn, R. S. , Boomsma, D. I. , & Pol, H. E. H. (2012). White matter development in early puberty: A longitudinal volumetric and diffusion tensor imaging twin study. PLoS One, 7(4), e32316. 10.1371/journal.pone.0032316 22514599 PMC3326005

[jcv212061-bib-0009] Campbell‐Sills, L. , & Stein, M. B. (2007). Psychometric analysis and refinement of the connor–davidson resilience scale (CD‐RISC): Validation of a 10‐item measure of resilience. Journal of Traumatic Stress, 20(6), 1019–1028. 10.1002/jts.20271 18157881

[jcv212061-bib-0010] Chahal, R. , Delevich, K. , Kirshenbaum, J. S. , Borchers, L. R. , Ho, T. C. , & Gotlib, I. H. (2021). Sex differences in pubertal associations with fronto‐accumbal white matter morphometry: Implications for understanding sensitivity to reward and punishment. NeuroImage, 226, 117598. 10.1016/j.neuroimage.2020.117598 33249215 PMC7840818

[jcv212061-bib-0011] Chahal, R. , Kirshenbaum, J. S. , Miller, J. G. , Ho, T. C. , & Gotlib, I. H. (2021). Higher executive control network coherence buffers against puberty‐related increases in internalizing symptoms during the COVID‐19 pandemic. Biological Psychiatry. Cognitive Neuroscience and Neuroimaging, 6(1), 79–88. 10.1016/j.bpsc.2020.08.010 33097469 PMC7455201

[jcv212061-bib-0012] Cohen, S. , Kamarck, T. , & Mermelstein, R. (1983). A global measure of perceived stress. Journal of Health and Social Behavior, 24(4), 385–396. 6668417

[jcv212061-bib-0013] Colich, N. L. , Williams, E. S. , Ho, T. C. , King, L. S. , Humphreys, K. L. , Price, A. N. , Ordaz, S. J. , & Gotlib, I. H. (2017). The association between early life stress and prefrontal cortex activation during implicit emotion regulation is moderated by sex in early adolescence. Development and Psychopathology, 29(5), 1851–1864. 10.1017/S0954579417001444 29162186 PMC5726300

[jcv212061-bib-0014] Connor, K. M. , & Davidson, J. R. T. (2003). Development of a new resilience scale: The connor‐davidson resilience scale (CD‐RISC). Depression and Anxiety, 18(2), 76–82. 10.1002/da.10113 12964174

[jcv212061-bib-0015] Davidovich, S. , Collishaw, S. , Thapar, A. K. , Harold, G. , Thapar, A. , & Rice, F. (2016). Do better executive functions buffer the effect of current parental depression on adolescent depressive symptoms? Journal of Affective Disorders, 199, 54–64. 10.1016/j.jad.2016.03.049 27085164 PMC4871808

[jcv212061-bib-0016] Diamond, A. (2013). Executive functions. Annual Review of Psychology, 64(1), 135–168. 10.1146/annurev-psych-113011-143750 PMC4084861 23020641

[jcv212061-bib-0017] Fernández‐Guasti, A. , Fiedler, J. L. , Herrera, L. , & Handa, R. J. (2012). Sex, stress, and mood disorders: At the intersection of adrenal and gonadal hormones. Hormone and Metabolic Research = Hormon‐ Und Stoffwechselforschung = Hormones Et Metabolisme, 44(8), 607–618. 10.1055/s-0032-1312592 22581646 PMC3584173

[jcv212061-bib-0018] Frye, R. E. , Hasan, K. , Malmberg, B. , deSouza, L. , Swank, P. , Smith, K. , & Landry, S. (2010). Superior longitudinal fasciculus and cognitive dysfunction in adolescents born preterm and at term. Developmental Medicine and Child Neurology, 52(8), 760–766. 10.1111/j.1469-8749.2010.03633.x 20187879 PMC2910222

[jcv212061-bib-0019] Gaillard, A. , Fehring, D. J. , & Rossell, S. L. (2021). Sex differences in executive control: A systematic review of functional neuroimaging studies. European Journal of Neuroscience, 53(8), 2592–2611. 10.1111/ejn.15107 33423339

[jcv212061-bib-0020] Grissom, N. M. , & Reyes, T. M. (2019). Let’s call the whole thing off: Evaluating gender and sex differences in executive function. Neuropsychopharmacology, 44(1), 86–96. 10.1038/s41386-018-0179-5 30143781 PMC6235899

[jcv212061-bib-0021] Hamilton, J. L. , Stange, J. P. , Abramson, L. Y. , & Alloy, L. B. (2015). Stress and the development of cognitive vulnerabilities to depression explain sex differences in depressive symptoms during adolescence. Clinical Psychological Science, 3(5), 702–714. 10.1177/2167702614545479 26509106 PMC4617303

[jcv212061-bib-0022] Helsel, W. J. , & Matson, J. L. (1984). The assessment of depression in children: The internal structure of the Child Depression Inventory (CDI). Behaviour Research and Therapy, 22(3), 289–298. 10.1016/0005-7967(84)90009-3 6466279

[jcv212061-bib-0023] Hodes, G. E. , & Epperson, C. N. (2019). Sex differences in vulnerability and resilience to stress across the life span. Biological Psychiatry, 86(6), 421–432. 10.1016/j.biopsych.2019.04.028 31221426 PMC8630768

[jcv212061-bib-0024] Jalbrzikowski, M. , Liu, F. , Foran, W. , Calabro, F. , Roeder, K. , Devlin, B. , & Luna, B. (2019). Cognitive and default mode networks support developmental stability in functional connectome fingerprinting through adolescence. BioRxiv. 10.1101/812719

[jcv212061-bib-0025] Jenkinson, M. , Beckmann, C. F. , Behrens, T. E. J. , Woolrich, M. W. , & Smith, S. M. (2012). FSL. NeuroImage, 62(2), 782–790. 10.1016/j.neuroimage.2011.09.015 21979382

[jcv212061-bib-0026] Joyce, S. , Shand, F. , Tighe, J. , Laurent, S. J. , Bryant, R. A. , & Harvey, S. B. (2018). Road to resilience: A systematic review and meta‐analysis of resilience training programmes and interventions. BMJ Open, 8(6), e017858. 10.1136/bmjopen-2017-017858 PMC6009510 29903782

[jcv212061-bib-0027] Juraska, J. M. , & Willing, J. (2017). Pubertal onset as a critical transition for neural development and cognition. Brain Research, 1654, 87–94. 10.1016/j.brainres.2016.04.012 27060769 PMC5053848

[jcv212061-bib-0028] Kaufman, J. , Birmaher, B. , Brent, D. , Rao, U. , Flynn, C. , Moreci, P. , Williamson, D. , & Ryan, N. (1997). Schedule for affective disorders and Schizophrenia for school‐age children‐present and Lifetime version (K‐SADS‐PL): Initial reliability and validity data. Journal of the American Academy of Child & Adolescent Psychiatry, 36(7), 980–988. 10.1097/00004583-199707000-00021 9204677

[jcv212061-bib-0029] Kessler, R. C. (1997). The effects of stressful life events on depression. Annual Review of Psychology, 48(1), 191–214. 10.1146/annurev.psych.48.1.191 9046559

[jcv212061-bib-0030] King, L. S. , Humphreys, K. L. , Camacho, M. C. , & Gotlib, I. H. (2019). A person‐centered approach to the assessment of early life stress: Associations with the volume of stress‐sensitive brain regions in early adolescence. Development and Psychopathology, 31(2), 643–655. 10.1017/S0954579418000184 29716668 PMC6214790

[jcv212061-bib-0031] Lee, E.‐H. (2012). Review of the psychometric evidence of the perceived stress scale. Asian Nursing Research, 6(4), 121–127. 10.1016/j.anr.2012.08.004 25031113

[jcv212061-bib-0032] Long, J. A. (2021). Interactions: Comprehensive, user‐friendly toolkit for probing interactions. Version 1.1.5. https://CRAN.R‐project.org/package=interactions

[jcv212061-bib-0033] Marečková, K. , Klasnja, A. , Andrýsková, L. , Brázdil, M. , & Paus, T. (2019). Developmental origins of depression‐related white matter properties: Findings from a prenatal birth cohort. Human Brain Mapping, 40(4), 1155–1163. 10.1002/hbm.24435 30367731 PMC6865745

[jcv212061-bib-0034] Morris, N. M. , & Udry, J. R. (1980). Validation of a self‐administered instrument to assess stage of adolescent development. Journal of Youth and Adolescence, 9(3), 271–280. 10.1007/BF02088471 24318082

[jcv212061-bib-0035] Niendam, T. A. , Laird, A. R. , Ray, K. L. , Dean, Y. M. , Glahn, D. C. , & Carter, C. S. (2012). Meta‐analytic evidence for a superordinate cognitive control network subserving diverse executive functions. Cognitive, Affective, & Behavioral Neuroscience, 12(2), 241–268. 10.3758/s13415-011-0083-5 PMC3660731 22282036

[jcv212061-bib-0036] Pan, F. , Xu, Y. , Zhou, W. , Chen, J. , Wei, N. , Lu, S. , Shang, D. , Wang, J. , & Huang, M. (2020). Disrupted intrinsic functional connectivity of the cognitive control network underlies disease severity and executive dysfunction in first‐episode, treatment‐naive adolescent depression. Journal of Affective Disorders, 264, 455–463. 10.1016/j.jad.2019.11.076 31780136

[jcv212061-bib-0037] R Core Team . (2017). R: a language and environment for statistical computing. R Foundation for Statistical Computing. Retrieved from https://www.R‐project.org/

[jcv212061-bib-0038] Raffelt, D. A. , Tournier, J.‐D. , Smith, R. E. , Vaughan, D. N. , Jackson, G. , Ridgway, G. R. , & Connelly, A. (2017). Investigating white matter fibre density and morphology using fixel‐based analysis. NeuroImage, 144(Pt A), 58–73. 10.1016/j.neuroimage.2016.09.029 27639350 PMC5182031

[jcv212061-bib-0039] Salk, R. H. , Hyde, J. S. , & Abramson, L. Y. (2017). Gender differences in depression in representative national samples: Meta‐analyses of diagnoses and symptoms. Psychological Bulletin, 143(8), 783–822. 10.1037/bul0000102 28447828 PMC5532074

[jcv212061-bib-0040] Saylor, C. F. , Finch, A. J. , Spirito, A. , & Bennett, B. (1984). The children’s depression inventory: A systematic evaluation of psychometric properties. Journal of Consulting and Clinical Psychology, 52(6), 955–967. 10.1037/0022-006X.52.6.955 6520288

[jcv212061-bib-0041] Schermuly, I. , Fellgiebel, A. , Wagner, S. , Yakushev, I. , Stoeter, P. , Schmitt, R. , Knickenberg, R. J. , Bleichner, F. , & Beutel, M. E. (2010). Association between cingulum bundle structure and cognitive performance: An observational study in major depression. European Psychiatry: The Journal of the Association of European Psychiatrists, 25(6), 355–360. 10.1016/j.eurpsy.2010.05.001 20621455

[jcv212061-bib-0042] Sherman, L. E. , Rudie, J. D. , Pfeifer, J. H. , Masten, C. L. , McNealy, K. , & Dapretto, M. (2014). Development of the default mode and central executive networks across early adolescence: A longitudinal study. Developmental Cognitive Neuroscience, 10, 148–159. 10.1016/j.dcn.2014.08.002 25282602 PMC4854607

[jcv212061-bib-0043] Shields, G. S. , Sazma, M. A. , & Yonelinas, A. P. (2016). The effects of acute stress on Core executive functions: A meta‐analysis and comparison with cortisol. Neuroscience & Biobehavioral Reviews, 68, 651–668. 10.1016/j.neubiorev.2016.06.038 27371161 PMC5003767

[jcv212061-bib-0044] Shirtcliff, E. A. , Dahl, R. E. , & Pollak, S. D. (2009). Pubertal development: Correspondence between hormonal and physical development. Child Development, 80(2), 327–337. 10.1111/j.1467-8624.2009.01263.x 19466995 PMC2727719

[jcv212061-bib-0045] Timbremont, B. , Braet, C. , & Dreessen, L. (2004). Assessing depression in youth: Relation between the children’s depression inventory and a structured interview. Journal of Clinical Child and Adolescent Psychology, 33(1), 149–157. 10.1207/S15374424JCCP3301_14 15028549

[jcv212061-bib-0046] Tournier, J.‐D. , Smith, R. E. , Raffelt, D. A. , Tabbara, R. , Dhollander, T. , Pietsch, M. , Christiaens, D. , Jeurissen B. , Yeh C.‐H. , & Connelly A. (2019). MRtrix3: A fast, flexible and open software framework for medical image processing and visualisation [Preprint]. Neuroscience. 10.1101/551739 31473352

[jcv212061-bib-0047] Twenge, J. M. (2020). Increases in depression, self‐Harm, and suicide among U.S. Adolescents after 2012 and links to technology use: Possible mechanisms. Psychiatric Research and Clinical Practice, 2(1), 19–25. 10.1176/appi.prcp.20190015 PMC9176070 36101887

[jcv212061-bib-0048] Vestergaard, M. , Madsen, K. S. , Baaré, W. F. C. , Skimminge, A. , Ejersbo, L. R. , Ramsøy, T. Z. , Gerlach, C. , Akeson P. , Paulson O. B. , & Jernigan T. L. (2011). White matter microstructure in superior longitudinal fasciculus associated with spatial working memory performance in children. Journal of Cognitive Neuroscience, 23(9), 2135–2146. 10.1162/jocn.2010.21592 20964591

[jcv212061-bib-0049] Vlasova, R. M. , Siddarth, P. , Krause, B. , Leaver, A. M. , Laird, K. T. , St Cyr, N. , Lavretsky, H. , & Lavretsky, H. (2018). Resilience and white matter integrity in geriatric depression. American Journal of Geriatric Psychiatry, 26(8), 874–883. 10.1016/j.jagp.2018.04.004 PMC6086733 29803529

[jcv212061-bib-0050] Wang, S. , Yang, C. , Zhao, Y. , Lai, H. , Zhang, L. , & Gong, Q. (2020). Sex‐linked neurofunctional basis of psychological resilience in late adolescence: A resting‐state functional magnetic resonance imaging study. European Child & Adolescent Psychiatry, 29(8), 1075–1087. 10.1007/s00787-019-01421-6 31641900

[jcv212061-bib-0051] Wheelock, M. D. , Goodman, A. M. , Harnett, N. G. , Wood, K. H. , Mrug, S. , Granger, D. A. , & Knight, D. C. (2021). Sex‐related differences in stress reactivity and cingulum white matter. Neuroscience, 459, 118–128. 10.1016/j.neuroscience.2021.02.014 33588003 PMC7965343

[jcv212061-bib-0052] Yoon, T. , Keller, M. L. , De‐Lap, B. S. , Harkins, A. , Lepers, R. , & Hunter, S. K. (2009). Sex differences in response to cognitive stress during a fatiguing contraction. Journal of Applied Physiology, 107(5), 1486–1496. 10.1152/japplphysiol.00238.2009 19729594 PMC2777799

